# Effect of confinement potential shape on the electronic, thermodynamic, magnetic and transport properties of a GaAs quantum dot at finite temperature

**DOI:** 10.1038/s41598-019-52190-w

**Published:** 2019-11-01

**Authors:** K. Luhluh Jahan, Bahadir Boyacioglu, Ashok Chatterjee

**Affiliations:** 1 School of Physics, University of Hyderabad, Hyderabad, 500 046 India; 2 Vocational School of Health Services, Ankara University, 06290 Kecioren, Ankara Turkey

**Keywords:** Quantum dots, Theoretical physics

## Abstract

The effect of the shape of the confinement potential on the electronic, thermodynamic, magnetic and transport properties of a GaAs quantum dot is studied using the power-exponential potential model with steepness parameter *p*. The average energy, heat capacity, magnetic susceptibility and persistent current are calculated using the canonical ensemble approach at low temperature. It is shown that for soft confinement, the average energy depends strongly on *p* while it is almost independent of *p* for hard confinement. The heat capacity is found to be independent of the shape and depth of the confinement potential at low temperatures and for the magnetic field considered. It is shown that the system undergoes a paramagnetic-diamagnetic transition at a critical value of the magnetic field. It is furthermore shown that for low values of the potential depth, the system is always diamagnetic irrespective of the shape of the potential if the magnetic field exceeds a certain value. For a range of the magnetic field, there exists a window of *p* values in which a re-entrant behavior into the diamagnetic phase can occur. Finally, it is shown that the persistent current in the present quantum dot is diamagnetic in nature and its magnitude increases with the depth of the dot potential but is independent of *p* for the parameters considered.

## Introduction

The subject of quantum dots (QDs) has attracted unprecedented attention for its fundamental appeal and technological potential^1^. One of the most important advantages with quantum dots is that its shape and size can be controlled according to the desired properties. It is, of course, essential to know the nature of the confinement potential to formulate any theory of quantum dots. Early experiments indicated that the confinement potential in a quantum dot is essentially harmonic. Consequently, a large body of literature has piled up on the subject of parabolic quantum dots^2–16^. However, some recent experiments have suggested that the confinement potential in a quantum dot is rather anharmonic and has a finite depth^17,18^. Adamowsky *et al*.^17^ suggested an attractive Gaussian potential model for confinement which has been found to be more realistic. Subsequently, extensive investigaons^19–37^ have been made on several properties of QDs using the Gaussian confinement potential. Ciurla *et al*.^38^ have studied the problem of confinement potential profile in QDs and proposed a new class of confinement potentials, called the power-exponential (PE) potentials, which are sufficiently flexible to approximate the realistic confinement potentials in the QDs. They showed that the commonly used model confinement potentials, i.e. the parabolic and rectangular potential wells, could be obtained as the limiting forms of the power-exponential. Kwaśniowski and Adamowski^39^ have studied the exchange interaction for electrons in coupled QDs by a configuration interaction method using confinement potentials with different profiles. The photoionization of a donor impurity in a power-exponential quantum dot (PEQD) has been studied by Xie^40^ by using the PE potential model and the results have been presented as a function of the diffusion photon energy. It has been shown that the Photoionization Cross Section of a donor impurity in a QD is strongly dependent on the shape of the PE potentials, the geometrical size, and the impurity ion position.

To our knowledge, no investigation has so far been made on the temperature-dependent electronic, thermodynamic, magnetic and transport properties of a PEQD. In the present paper, we shall make an attempt in this direction in the presence of the spin-Zeeman interaction.

## Model

**Figure 1:**
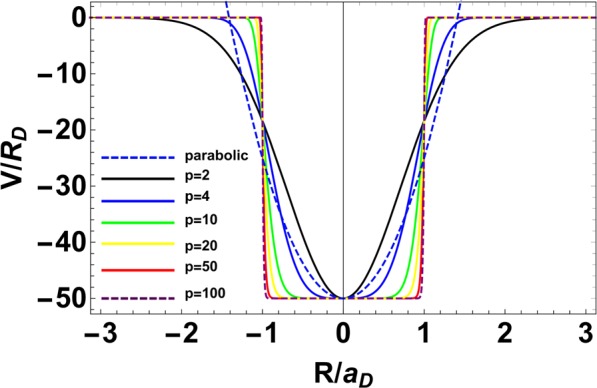
The shape of parabolic and the power-exponential confinement potential for *p* = 2, 4, 10, 20, 50 and 100. The donor Bohr Radius *a*_*D*_ is s the unit of length and the donor Rydberg *R*_*D*_ is the unit of energy^38^.

The Hamiltonian of a system of an electron moving in a two-dimensional (2D) confining potential (***ρ***) in the presence of an external magnetic field, ***B*** may be written as

1 $$H=\frac{1}{2{m}^{\ast }}{({\boldsymbol{p}}+\frac{e}{c}{\boldsymbol{A}})}^{2}+V({\boldsymbol{\rho }}),$$

where ***ρ*** refers to the position vector of an electron in two dimensions, ***p*** is the corresponding momentum operator, m* is the electron effective mass and ***A*** is the vector potential corresponding to the magnetic field ***B*** which has been applied in the z direction. Choosing the gauge of **A** as ***A*** = (−*By*/2, *Bx*/2, 0) such that ***A*** is divergence-less and including the spin-Zeeman term, we can write Hamiltonian (1) as

2 $$H=-\,\frac{{\hslash }^{2}}{2{m}^{\ast }}{\nabla }_{\rho }^{2}+V({\boldsymbol{\rho }})+\frac{1}{8}{m}^{\ast }{\omega }_{c}^{2}{\rho }^{2}+\frac{1}{2}\hslash {\omega }_{c}({\hat{L}}_{z}+{g}^{\ast }{\hat{S}}_{z}),$$

where *L*_*z*_ is the z-component of the angular momentum of the electron, *ω*_*c*_ is the bare cyclotron frequency given by *ω*_*c*_ = *eB*/m* and *V*(***ρ***) is the spherically symmetric PE potential^38^ given by

3 $$V({\boldsymbol{\rho }})=-\,{V}_{0}{e}^{-{(\frac{\rho }{R})}^{p}},$$

where *V*_0_ denotes the depth of the potential, *R* gives a measure of the range of the potential and thus represents the effective confinement length or the size of the QD and the parameter *p* decides the shape of the confinement potential and gives a measure of the steepness of the potential at the QD boundary. The smaller (larger) is the value *p*, the softer (harder) is the potential. For *p* = 2, the confinement potential has the Gaussian shape. This potential is soft, that is the potential has a fairly small steepness at the QD boundary. For *p* ≥ 4 the confinement potential becomes hard that is the potential at the QD boundary becomes very steep. For *p* ≥10, one deals with a very hard, rectangular-type confinement potential (Fig. 1).

## Formulation

We assume that the deviation of the shape of the PE potentials from the parabolic potential is small enough so that it can be treated as a parabolic potential plus a perturbation. This is a reasonable assumption for small *r* and since in a QD, *r* would be generally small, it can be considered as a fairly good approximation. So we rewrite the Hamiltonian (1) as

4 $$H={H}_{0}+{H}_{1},$$

with

5 $${H}_{0}=-\,\frac{{\hslash }^{2}}{2{m}^{\ast }}{{\boldsymbol{\nabla }}}_{\rho }^{2}+\frac{1}{2}{m}^{\ast }{\tilde{\omega }}^{2}{\rho }^{2}+\frac{1}{2}\hslash {\omega }_{c}({\hat{L}}_{z}+{g}^{\ast }{\hat{S}}_{z})-{V}_{0},$$

6 $${H}_{1}=-\,\lambda [\frac{1}{2}{m}^{\ast }{\omega }_{h}^{2}{\rho }^{2}+{V}_{0}({e}^{-{(\frac{\rho }{R})}^{p}}-1)],$$

where

7 $${\tilde{\omega }}^{2}={\omega }_{h}^{2}+\frac{{\omega }_{c}^{2}}{4},\,{\omega }_{h}^{2}=\frac{{V}_{0}}{{m}^{\ast }{R}^{2}},$$

and *λ* = 0 for a parabolic confinement and *λ* = 1 for the PE potential. We assume that the sole effect of *H*_1_ is to renormalize the frequency $\tilde{\omega }$. So we treat *H*_1_ at the mean field level. More specifically we write *H*_1_ as

8 $${H}_{1}=\lambda [\frac{{V}_{0}}{\langle {\rho }^{2}\rangle }-\frac{1}{2}{m}^{\ast }{\omega }_{h}^{2}-{V}_{0}\frac{\langle {e}^{-{(\frac{\rho }{R})}^{p}}\rangle }{\langle {\rho }^{2}\rangle }]{\rho }^{2},$$

where 〈*ρ*^2^〉 is the expectation value of *ρ*^2^ with respect to the wave function of the harmonic oscillator of frequency $\tilde{\omega }$. The problem now reduces to an effective parabolic problem described by the Hamiltonian

9 $$H=-\,\frac{{\hslash }^{2}}{2{m}^{\ast }}{\nabla }_{\rho }^{2}+\frac{1}{2}{m}^{\ast }{\omega }^{2}{\rho }^{2}+\frac{1}{2}\hslash {\omega }_{c}({\hat{L}}_{z}+{g}^{\ast }{\hat{S}}_{z})-{V}_{0},$$

where *ω* is the effective frequency and g* is the effective Lande-g factor (which is equal to −0.44 for GaAs). The effective problem now satisfies the Schrödinger equation

10 $$H\,{\Psi }_{nls}(\rho ,\theta ,\sigma )={E}_{nls}{\Psi }_{nls}(\rho ,\theta ,\sigma )\,,$$

where Ψ_*nls*_(*ρ*, *θ*, *σ*) is the wave function given by the Fock–Darwin states^41,42^

11 $${\Psi }_{nls}(\rho ,\theta ,\sigma )=\sqrt{\frac{{\alpha }^{2}n!}{(n+|l|)!\pi }}{(\alpha \rho )}^{|l|}{L}_{n}^{|l|}({\alpha }^{2}{\rho }^{2}){e}^{-\frac{{\alpha }^{2}}{2}{\rho }^{2}+il\theta }{\chi }_{s}(\sigma ),$$

where *α* = (*m* * *ω*/$\hslash$)^1/2^, *n* is the radial quantum number, *l*(= 0, ±1, ±2, …) is the azimuthal angular momentum quantum number, ${L}_{n}^{|l|}$ is the associated Laguerre polynomial, *χ*_*s*_*(σ*) is the eigenstate of the spin operator with eigenvalue *s* = ±(1/2) and *E*_*nls*_ is given by^41,42^

12 $${E}_{nls}=(2n+|l|+1)\hslash \omega +\frac{1}{2}\hslash {\omega }_{c}(l+{g}^{\ast }s)-{V}_{0}.$$

At zero temperature, the magnetization and the magnetic susceptibility of a system in a state (*n*, *l)* are defined by

13 $${M}_{nl}(B)=-\,\frac{\partial {E}_{nl}}{\partial B};\,\chi =\frac{\partial M}{\partial B}$$

The partition function for the present system can be exactly calculated and is given by

14 $$Z(T,\,B)={\sum }_{n=0}^{\infty }{\sum }_{l=0}^{\pm \infty }{\sum }_{s=-1/2}^{s=1/2}{e}^{-\beta {E}_{nls}}$$

where *β* = 1/*k*_*B*_*T*. The partition function can be used to obtain the average energy, magnetization, magnetic susceptibility and heat capacity as

15 $$\langle E\rangle =-\,\frac{1}{Z}(\frac{\partial Z}{\partial \beta });\langle M\rangle =\frac{1}{\beta Z}(\frac{\partial Z}{\partial B});\chi =\frac{\partial \langle M\rangle }{\partial B};\,C=\frac{\partial \langle E\rangle }{\partial T}.$$

We are also interested in the temperature dependent persistent current and therefore we calculate the canonical ensemble average of ${J}_{nl}^{spin}$ as follows:

16 $$\langle {J}_{nl}^{spin}\rangle =\frac{{\sum }_{n,l,s}\,{J}_{nl}^{spin}\,{e}^{-\beta {E}_{nls}}}{{\sum }_{n,l,s}\,{e}^{-\beta {E}_{nls}}}.$$

## Numerical Results and Discussion

**Figure 2:**
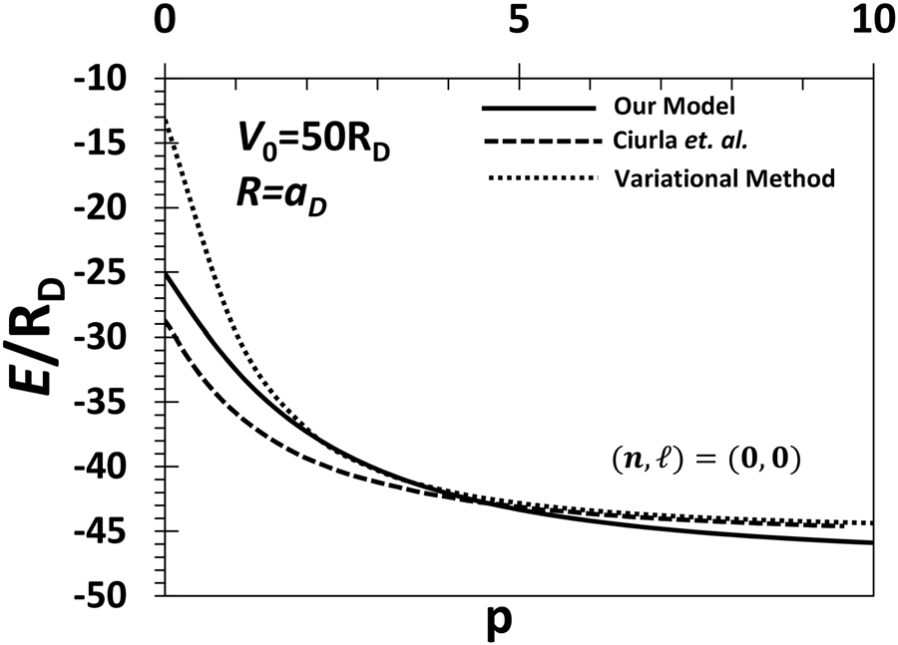
Ground state energy of a QD with the power-exponential potential as a confinement potential as a function of the shape of the potential (*p*) in the absence of the magnetic field with *V*_0_ = 50R_D_ and *R* = a_D_.

Before we present our results on magnetization and susceptibility, it may be worthwhile to show that the approximation made in Eq. (8) for *H*_1_ is fairly good. To that end, we shall compare our present results (with B = 0) for the ground state energy with the ones obtained by the Ritz variational calculation performed by us and with those of Ciurla *et al*.^38^ obtained using the high-order finite-difference method. Figure 2 shows the comparison for different values of *p* with *V*_0_ = 50*R*_*D*_ and *R* = *a*_*D*_. It is clearly evident that our results are better than the variational results and are in good agreement with those of Ciurla *et al*.^38^. This imparts a fair amount of confidence in our approximation for the ground state calculation. However, our approximation turns out to be not so good for the excited states.

**Figure 3:**
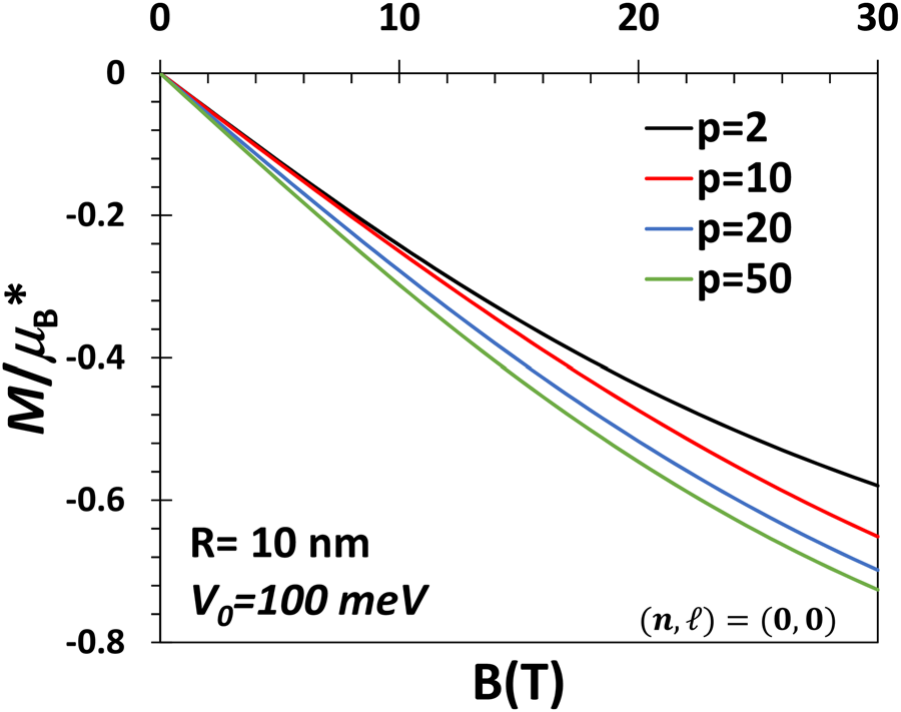
*M* vs. B for the GS of a QD with *V*_0_ = 100 meV, *R* = 10 nm and p = 2, 10, 20 and 50.

**Figure 4:**
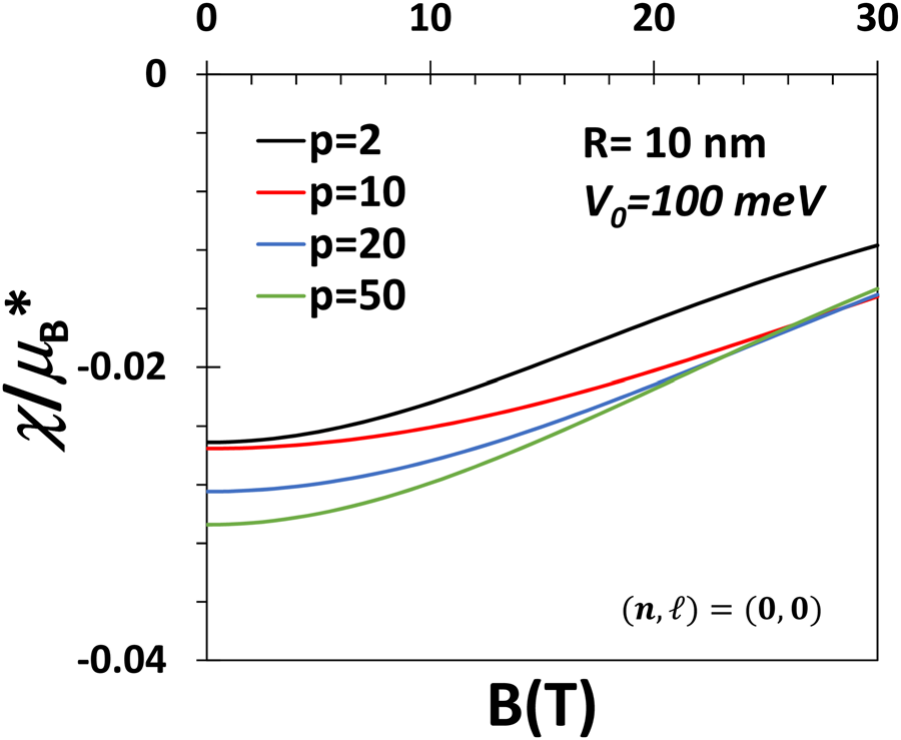
*χ* vs *B* for the GS of a QD with *V*_0_ = 100 meV, *R* = 10 nm and 50 for p = 2, 10, 20 and 50.

In Fig. 3 we show the variation of magnetization for GaAs PEQD um dot as a function of the magnetic field for PE potentials for the ground state. The first observation we make here is that the magnitude of magnetization (|**M|)** in a QD increases with the increase in the magnetic field for a given value of *V*_0_ and *R* for PE potentials. This is of course understandable. Also one can see the system is always in a diamagnetic state. This is again understandable in view of the inherent diamagnetism of the electron in a potential and the absence of paramagnetism in the GS. |**M|** increases with the applied magnetic field which is again an expected behavior. The figure also shows that as the magnetic field increases, |**M**| increases with *p*. Figure 4 shows the behavior of the susceptibility as a function of the magnetic field.

**Figure 5:**
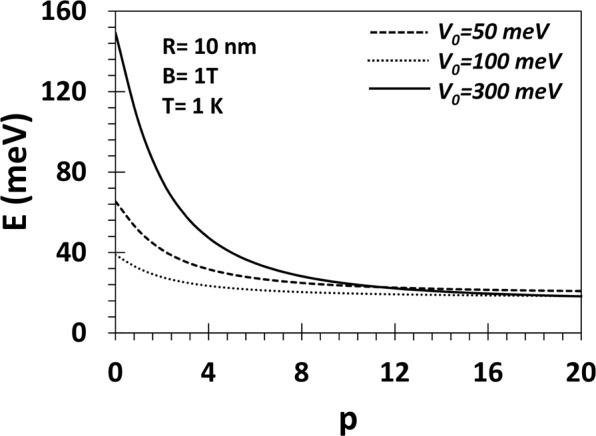
*E* vs p for a GaAs QD with *V*_0_ = 50, 100, 300 meV, *R* = 10 nm, *B* = 1*T* and, *T* = 1 *K*.

In Fig. 5 we plot the average thermal energy (〈*E*〉) of an electron in a GaAs QD as a function of the parameter *p* for *B* = 1*T*, *T* = 1 *K*, *R* = 10 *nm* and for three values of *V*_0_. One may observe that 〈*E*〉 increases monotonically as *p* decreases. Thus the average energy is higher if the potential is softer. The increase in 〈*E*〉 with decreasing *p* is sharp and substantial for small *p* and large *V*_0_. This is because as the potential becomes softer, it can accommodate a larger number of bound states at the Rydberg levels leading to a larger 〈*E*〉.

**Figure 6:**
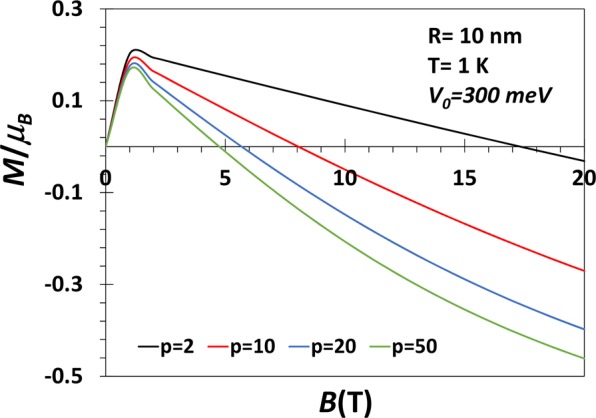
*M* vs B for a GaAs QD with *V*_0_ = 300 meV, *R* = 10 nm, *B* = 1*T* and *p* = 2, 10, 20, 50 at *T* = 1 *K*.

**Figure 7:**
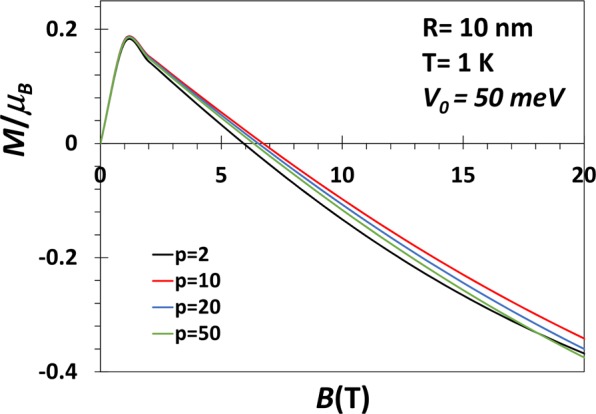
*M* vs *B* for a GaAs QD with *V*_0_ = 50 meV, *R* = 10 nm, *B* = 1*T* and *p* = 2, 10, 20, 50 at *T* = 1 *K*.

**Figure 8:**
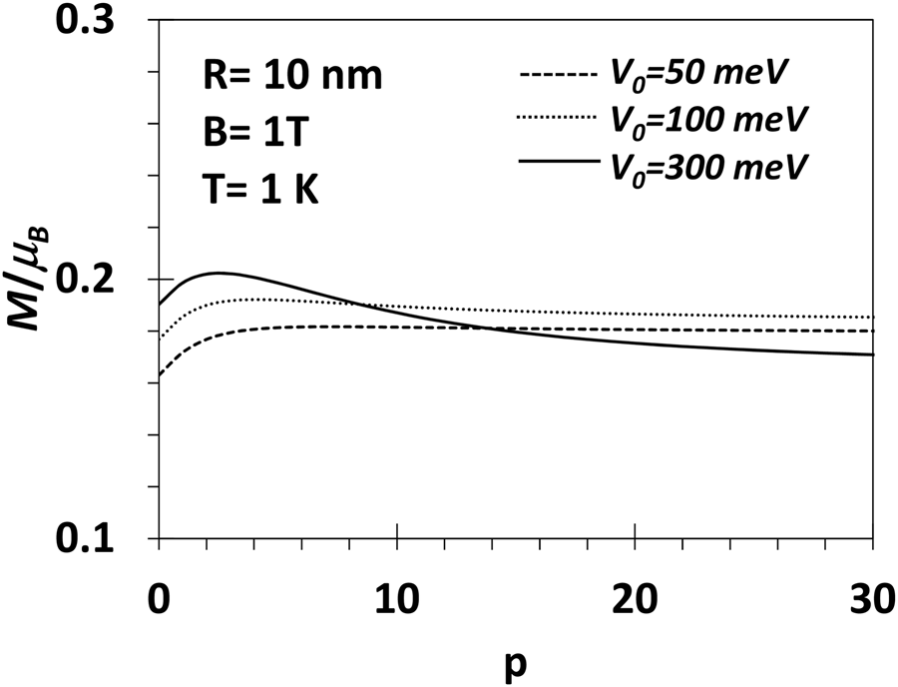
*M* vs *p* for a GaAs QD with *V*_0_ = 50, 100, 300 *meV*, *R* = 10 *nm*, *B* = 1*T*.

Figure 6 shows the variation of the average magnetization of a GaAs QD with *V*_0_ = 300 meV as a function of *B* for several values of *p*at *T* = 1 *K*. 〈*M*〉 initially increases with *B*, reaches a maximum at a certain value of *B* and at low values of *B*, 〈*M*〉 is essentially independent of *p*. Above a certain value of *B*, 〈*M*〉 decreases with a further increase in *B* and becomes negative and continues to become more and more negative with increasing *B*. The behaviour is qualitatively for different *p* values. Quantitatively, however, as *p* increases, 〈*M*〉 becomes negative at lower values of *B* and thus attains a larger negative value for the same value of *B*. In Fig. 7, we study the same 〈*M*〉 vs *B* graph for *V*_0_ = 50 meV. It is interesting to see that for a shallow quantum dot, the shape does not play much significant role. This is also an expected behaviour. In Fig. 8, we show the variation of the average magnetization (〈*M*〉/〈*μ*〉_*B*_) directly as a function of the parameter *p* for a GaAs QD with *R* = 10 *nm*, *V*_0_ = 50, 100, 300 meV and *B* = 1 *T* and *T*=1 K. The magnetization remains positive all through in conformity with Fig. 7. Furthermore, at small *p*, 〈*M*〉 increases with *p*, attains a maximum and then decreases with the further increase in *p* and finally saturates to a constant value. When *p* becomes large, the confinement potential becomes very steep at the boundary mimicing a rectangular potential and in this case, only a few low-lying discrete states, contribute to the average energy because of the fast exponential decaying of the Boltzman factor for the higher excited states. This explains the saturation of the magnetization with *p*. The maximum in 〈*M*〉 tends to flatten out as *V*_0_ decreases. At small *p*, 〈*M*〉 increases with *V*_0_. This is because at small *p*, with increasing *V*_0_ many more Rydberg-like states are supported by the potential. Thus as *p* exceeds a certain value, for large *V*_0_, we observe a crossing behaviour.

**Figure 9:**
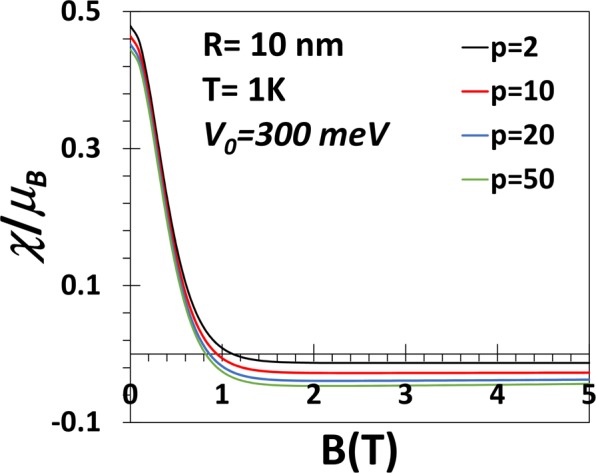
***χ*** vs *B* for a GaAs QD with *V*_0_ = 300 meV, *R* = 10 nm, *B* = 1*T* and *T* = 1 *K*.

**Figure 10:**
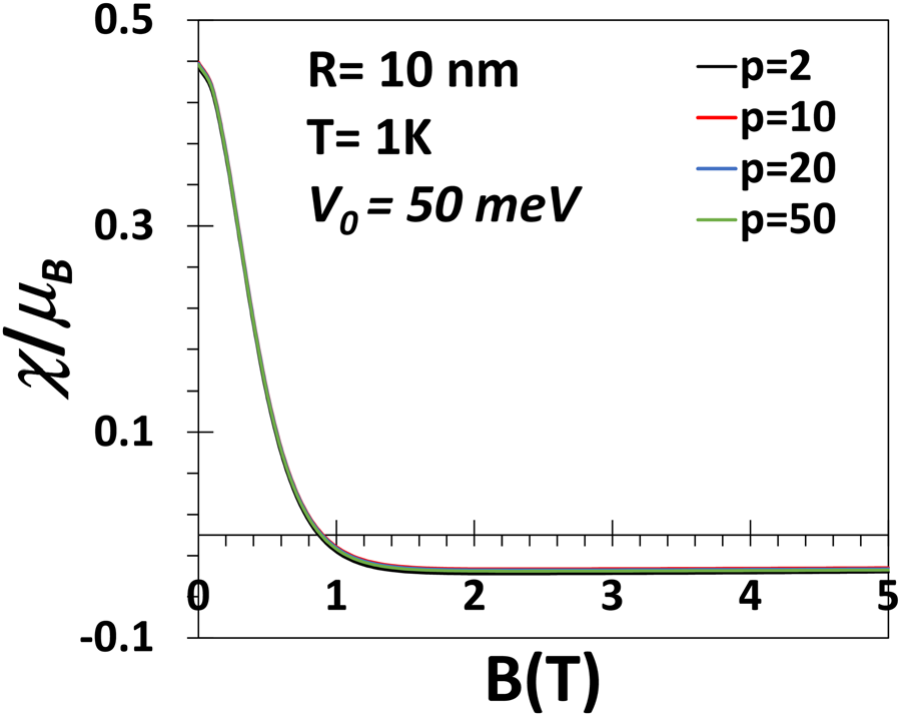
*χ* vs *B* for a GaAs QD with *V*_0_ = 50 meV, *R* = 10 *nm*, *p* = 2, 10, 20, 50 at *T* = 1 *K*.

In Fig. 9 we plot the magnetic susceptibility (*χ*) of a GaAs QD as a function of *B* for *p* = 2, 10, 20, 50 at *T* = 1 *K*. The figure shows that at very low magnetic field the susceptibility is paramagnetic for QDs of all geometries. As *B*increases, *χ* decreases and at a certain critical value of *B* which depends weakly on the value of the shape parameter, χ becomes diamagnetic. As *B* increases further, χ saturates to a constant diamagnetic value. ***χ*** vs *B* for a GaAs QD with *V*_0_ = 50 meV has been plotted in Fig. 10. For low *V*_0_, *χ* is essentially independent of *p* below approximately *B* = 1*T*. All these observations are consistent with Fig. 6.

**Figure 11:**
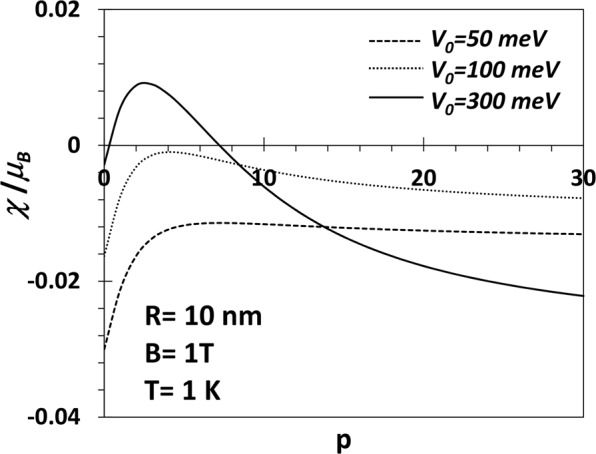
(*χ*/*μ*_*B*_) vs p of aGaAs QD with *V*_0_ = 50, 100, 300 meV, *R* = 10 nm, *B* = 1*T* and *T* = 1 *K*.

The explicit p-dependence of the susceptibility is shown in Fig. 11 at *B* = 1*T* for different values of *V*_0_ = 50 meV. *χ* is found to increase with *p* at small *p*, attains a maximum and then starts decreasing. For low values of *V*_0_, *χ* is diamagnetic for all values of *p*. However, for *V*_0_ = 300 meV, *χ* is negative for very small values of *p* and at a certain critical *p*, a transition occurs from the diamagnetic state to a paramagnetic state. Again *χ* attains a maximum value at a certain value of *p* beyond which it decreases monotonically and becomes negative above a certain *p*. So if *V*_0_ is made even larger, *χ* may be paramagnetic for a reasonable range of small values of *p* and then become diamagnetic at larger values of *p*.Thus we can have a re-entrant behaviour in the diamagnetic phase of a GaAs QD at low temperature and large *V*_0_.

**Figure 12:**
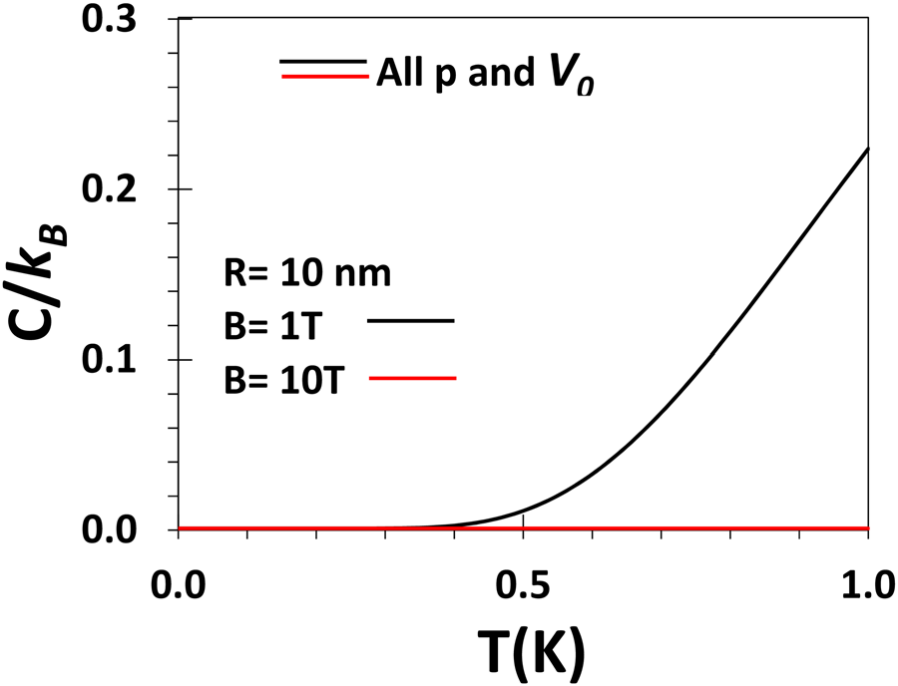
*C*/*k*_*B*_ vs T for B = 1 and 10 T for a GaAs QD with *R* = 10 *nm*, and different values of *p and V*_0_.

Figure 12 shows the behavior of heat capacity (*C*/*k*_*B*_) of a GaAs QD as a function of *T* for several values of *V*_0_ *and p* at *T* = 1 *K*. Clearly, the heat capacity is independent of *p* and *V*_0_. Though at high magnetic field, the heat capacity is zero at low temperature, at low magnetic field (*B* = 1 T), *C* is zero up to a certain value of *T* beyond which it increases with *T*. This can be easily explained from fundamental physics. At *B* = 1*T*, the system would require high energy to go to the excited states. At very low temperatures this energy is not available and therefore specific heat is expected to be zero. Nammas *et al*.^43^ have calculated the specific heat of a few-electron interacting QD using static fluctuation approximation and our result qualitatively agrees with their result.

**Figure 13:**
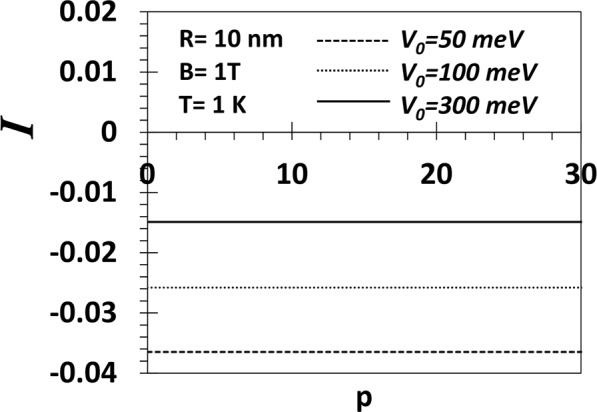
Persistent current (*I*) (*in unit of eω*_*h*_*/e*$\hslash$) vs *p* for *B* = 1 T, *T* = 1 K for a GaAs QD with *V*_0_ = 50, 100 and 300 meV.

In Fig. 13, we plot the average persistent current (*I*) as a function of *p* for *B* = 1, *T* = 1 for a GaAs QD with *R* = 10 nm and *V*_0_ = 50, 100 *and* 300 meV. We observe that at low temperature (*T* = 1 *K*), the persistent current is diamagnetic and its magnitude increases with decreasing *V*_0_. However the persistent current is independent of the shape of the confinement potential.

## Conclusions

In conclusion, we have studied in this work the effect of the shape of the confinement potential on the electronic, thermodynamic, magnetic and transport properties of a GaAs QD at low temperature using a power-exponential model for quantum confinement. We first observe that the GS energy decreases with the increase in *p* which is an expected behaviour as the potential flattens with increasing *p*. The GS magnetization turns out to be diamagnetic which is also an expected behaviour for a binding potential in the GS. In keeping with the above results, the GS diamagnetic susceptibility also increases with the magnetic field.

Since our approximation is not so good for higher excited states, we have calculated the thermodynamic quantities only at very low tempretature (at *T* = 1 *K*) because then, only the low-lying states will contribute. Our results show that even at low temperature the average energy decreases with increasing *p* at low *p* and saturates to a constant value as *p* increases. The average energy, however, increases with the increase in the depth of the potential *V*_0_ at small *p*, though at large *p*, the average energy becomes almost independent of *V*_0_. We have shown that at low temperature, the heat capacity of a GaAs QD is independent of the shape and depth of the confinement potential.

At *T* = 1 *K*, we have shown that *χ* is paramagnetic below a certain value of the magnetic field beyond which the system becomes diamagnetic. At small *V*_0_ and small *B* (less than 1*T*), *χ* is essentially independent of *p*. We have studied the variation of *χ* as a function of *p* explicitly at *T* = 1 *K*, *B* = 1*T* and for three values of *V*_0_. In all cases, *χ* increases with *p* at small *p*, attains a maximum and then starts decreasing. For low values of *V*_0_ (50, 100 meV), *χ*, however, remains always diamagnetic. For *V*_0_ = 300 meV, *χ* is diamagnetic at small *p*, becomes paramagnetic as *p* exceeds a certain critical value and finally again becomes as *p* exceeds another critical value. So if *V*_0_ is made still larger, *χ* may be paramagnetic even for small values of *p* and become diamagnetic at a larger value of *p*. Thus there exists a window of *p* values in which the re-entrant behaviour can show up. However, at very low temperature and large *p*, *χ* is never paramagnetic.

Finally we have shown that at low temperature, the persistent current in a GaAs QD is diamagnetic in nature and its magnitude increases with decreasing *V*_0_. It is however independent of *p* for the parameter values considered in this work.
